# Regulation of GVHD and GVL Activity via PD-L1 Interaction With PD-1 and CD80

**DOI:** 10.3389/fimmu.2018.03061

**Published:** 2018-12-21

**Authors:** Kaniel Cassady, Paul J. Martin, Defu Zeng

**Affiliations:** ^1^Irell and Manella Graduate School of Biological Sciences of City of Hope, Duarte, CA, United States; ^2^Department of Hematology/Hematopoietic Cell Transplantation, Beckman Research Institute at City of Hope National Medical Center, Duarte, CA, United States; ^3^Diabetes and Metabolism Research Institute, Beckman Research Institute, City of Hope National Medical Center, Duarte, CA, United States; ^4^Division of Clinical Research, Fred Hutchinson Cancer Research Center, Seattle, WA, United States; ^5^Department of Medicine, University of Washington, Seattle, WA, United States

**Keywords:** HCT, GVHD, alloreactive, T cell, PD-L1, PD-1, CD80

## Abstract

Allogeneic hematopoietic cell transplantation (HCT) is a curative therapy for hematological malignancies (i.e. leukemia and lymphoma), because graft-versus-leukemia (GVL) activity mediated by alloreactive T cells can eliminate residual malignant cells and prevent relapse. However, the same alloreactive T cells also mediate a severe side effect, graft-versus-host disease (GVHD), and prevention of GVHD while preserving GVL activity remains an elusive goal. The immune checkpoint molecule PD-L1 and its interaction with PD-1 receptor in regulating cancer immunity is under intensive and wide-spread study, but knowledge about this interaction in regulating GVHD and GVL activity is very limited. In this review, we summarize the literature exploring how PD-L1 interaction with its receptors PD-1 and CD80 regulate GVHD and GVL activities, how PD-L1 signaling regulates T cell metabolic profiles, and how a differential role of PD-L1 interaction with PD-1, CD80 or both may provide a novel avenue to prevent GVHD while preserving strong GVL effects.

## Introduction

Hematopoietic cell transplantation (HCT) provides curative therapy for hematological malignancies, such as lymphoma and leukemia, owing to graft-vs.-leukemia/lymphoma (GVL) effects mediated by alloreactive T cells (1–5). However, the same alloreactive T cells also mediate a severe side effect, called graft-vs.-host disease (GVHD). Prevention of GVHD while preserving GVL effect remains a long-sought and elusive goal.

Alloreactive T cells are activated by host antigen presenting cells (APCs) in lymphoid tissues early after HCT, and they then migrate to GVHD target tissues that were initially damaged by conditioning regimen to cause tissue inflammation and GVHD (6, 7). Acute GVHD is characterized by systemic lymphocyte infiltration and uncontrolled inflammation involving the release of damage-associated molecular pattern (DAMP) factors in target organs such as the gut, liver, lung, and skin (8–12). Chronic GVHD is a chronic autoimmune systemic syndrome characterized by moderate lymphocytic inflammation and fibrosis (13–16). Chronic GVHD and acute GVHD both affect the gut, liver, and skin, but chronic GVHD differs from acute GVHD in attacking other target tissue such as salivary and lacrimal glands and resembles a systemic auto-immune disease, like lupus, with dysregulated tolerance mechanisms (17–19).

GVHD pathogenesis is regulated by multiple factors, such as T regulatory CD4^+^ cells (Treg), donor APCs, B regulatory cells (Breg), myeloid-derived suppressor cells (MDSC), and immune checkpoints including interactions of programmed death ligand 1 (PD-L1) with programmed death-1 (PD-1) and CD80 (20–26). Although immune checkpoint regulation of tumor immunity is under intensive study (27), immune checkpoint regulation of GVHD and GVL effects, and especially their regulation by PD-L1 interaction with CD80, is not yet fully understood. In this review, we focus on how PD-L1 interaction with PD-1 and CD80 differentially regulates auto- and allo-immunity. We also discuss how these interactions can separate GVL activity from GVHD in preclinical animal models and highlight the recent clinical application and challenges of PD-L1/PD-1 blockade after HCT for augmenting GVL activity.

## Programmed Death Ligand-1 (PD-L1)-Mediated Signaling Pathways

PD-L1, (also known as CD274 or B7H1), is a member of the B7 family of immuno-coinhibitory and costimulatory molecules and functions as an immune checkpoint via its interaction with its receptors PD-1 and CD80 (also known as B7.1). PD-L1 is an Ig-V like transmembrane protein constitutively expressed by hematopoietic cells such as T, B and dendritic cells and by parenchymal cells in response to cytokine (i.e., IFN-γ) induction. PD-L1 was co-discovered in 2000 by two groups who reported it to have conflicting functions in the regulation of T cell activation, proliferation and apoptosis (28, 29). The Chen group first discovered and characterized the function of PD-L1 via cloning a homolog related to the B7.1 and B7.2 gene sequence, sequencing it and creating an expression plasmid containing the extracellular portion of PD-L1 in frame with the Fc portion of murine IgG2a (29). They found that costimulation of purified murine T cells in the presence of anti-CD3 and PD-L1-Ig led to an increase in T cell proliferation and IL-10 production, with moderate increase in IL-2 production—indicating a co-stimulatory signal mediated by the PD-L1 (29). On the other hand, costimulation of T cells with PD-L1-Ig produced by the Sharpe group led to a reduction in T cell proliferation and reduction in IL-10 production, indicating a coinhibitory signal mediated by PD-L1 (28).

Early *in vivo* studies on the role of PD-L1 were also conflicting. Tissue-specific transgenic expression of PD-L1 under the insulin promoter in islet beta cells augmented the rejection of islet grafts, which was associated with increased proliferation and reduced apoptosis of infiltrating CD8^+^ T cells (30). However, in a cardiac allograft model, treatment with PD-L1-Ig was associated with prolonged allograft survival and reduced lymphocytic infiltrate in the graft (7). Further characterization of the interactions of PD-L1/PD-1 and PD-L1/CD80 in unraveling the dual properties of the PD-L1-mediated signaling pathways are described below.

### PD-L1/PD-1 Signaling Pathway

The role of PD-L1 in regulating the immune response has been best characterized via its interaction with its dominant receptor PD-1, also termed Pdcd1 (6, 7, 23, 24). PD-1 is a monomeric co-inhibitory receptor that was originally identified in the 2B4.11 T cell hybridoma cell line as being upregulated upon induction of activation-induced apoptosis following stimulation with PMA and ionomycin (21). PD-1 expressed by activated T cells upon stimulation, is localized to the immunological synapse near the TCR and functions to attenuate T cell adaptive immune responses by inhibiting T cell proliferation and inducing T cell exhaustion, anergy, and apoptosis (6, 7). The importance of PD-1 in maintaining peripheral tolerance was highlighted by the generation of PD-1^−/−^ mice that develop Lupus-like arthritis and glomerulonephritis. Peripheral T and B cells from these mice exhibit hyper-reactivity upon stimulation (27, 31). The primary intracellular molecular mechanism responsible for PD-1 attenuation of the T cell response is attributed to the function of the immunoreceptor tyrosine-based inhibitory motif (ITIM) located in the cytoplasmic tail of PD-1 (7, 32). PD-L1/PD-1 ligation induces phosphorylation of this ITIM and recruits the protein-tyrosine phosphatases SHP1/2, in a TCR-stimulation dependent manner (33). Due to the proximity of the PD-1 cytoplasmic tail in the synapse to the TCR phosphorylation signaling cascade, SHP-1/2 phosphatase localization to PD-1 leads to dephosphorylation of TCR downstream signaling molecules, such as PI3K, ZAP70, and PTEN (34, 35). Collectively, dephosphorylation of this cascade leads to cell-cycle arrest, reduction in T cell proliferation/expansion and exhaustion/apoptosis, which can be reversed via PD-L1/PD-1 blockade to restore T cell function (36–38).

More recently, work by the Boussiotis group (39) has described a link between PD-L1/PD-1 signaling in the regulation of T cell metabolism by restricting nutrient uptake and utilization to inhibit T cell function (discussed below). Taken together, the PD-L1/PD-1 pathway inhibits the TCR signaling cascade to dampen the T cell immune response to maintain peripheral T cell tolerance.

### PD-L1/CD80 Signaling Pathway

In addition to interacting with PD-1, PD-L1 binds to and signals through a second receptor, CD80 (B7.1, B7-1). CD80, a member of the B7-super family, is a dimeric transmembrane protein, is constitutively expressed by T cells and is further upregulated upon T cell activation (22). Generally recognized for its function as a costimulatory ligand (along with CD86) for CD28, CD80 was first identified as a receptor on T cells for PD-L1 and was characterized by its ability to bidirectionally inhibit T cell responses (40, 41). The sites on PD-L1 that bind, respectively, to CD80 and PD-1 partially overlap, and the affinity of PD-L1 for CD80 is ~3-fold lower than its affinity for PD-1 (41). Using beads coated with anti-CD3 and CD80-Ig fusion protein or human IgG-Fc as a control, the authors stimulated CTLA4^−/−^ CD28^−/−^ T cells (T cells deficient for the two known binding partners of CD80). Under these conditions, costimulation with CD80-Ig decreased the proliferation of double-deficient T cells, indicating that CD80 can signal through PD-L1 expressed by T cells to inhibit proliferation (41). Furthermore, using beads coated with anti-CD3 and PD-L1-Ig fusion protein or human IgG-Fc as a control, the authors stimulated WT T cells and PD-1^−/−^ T cells. Under these conditions, costimulation with PD-L1-Ig decreased the proliferation of PD-1^−/−^ T cells, indicated that PD-L1 can signal through CD80 expressed by T cells to inhibit proliferation (41). Taken together, these results suggest a bi-directional inhibitory signal mediated by PD-L1/CD80 interaction.

*in vivo* studies using an anti-PD-L1 mAb that specifically blocks PD-L1/CD80 interaction while preserving PD-L1/PD-1 interaction have established PD-L1/CD80 “reverse signaling” into T cells as being pro-tolerogenic. In a murine model of immunization, blockade of PD-L1/CD80 interaction led to increased expansion and reduced induction of T cell anergy during the contraction phase following immunization (42). Moreover, in models of both Type-1 diabetes (T1D) and cardiac allograft transplantation, blockade of PD-L1/CD80 led to the increased production of proinflammatory cytokines by T cells and exacerbated T1D and graft rejection, respectively (43, 44).

On the other hand, agonistic anti-CD80 mAb interaction with naïve CD4^+^ T cells induced an increase in intracellular Ca2^+^ influx and led to phosphorylation of the Th1-promoting transcription factor T-bet (pT-bet). Phosphorylated T-bet localized to the *Ifng* locus and subsequently increased IFN-γ expression (45). Therefore, the impact of signaling mediated by PD-L1/CD80 interaction on T cell activation and tolerance induction requires further studies.

### PD-L1/CD28 Co-stimulatory Signaling Pathway Crosstalk

In addition to the direct effects of PD-L1 on T cell activation, expansion, and apoptosis exerted through its receptors PD-1 and CD80, PD-L1 signaling also exhibits cross-talk with another T cell co-stimulatory pathway, the CD28 signaling pathway. This canonical signaling pathway functions to complement the signals received via ligation of the TCR to activate the NF-kappaB transcriptional pathway and augment T cell survival and proliferation (46). Two groups recently co-discovered that the functional blockade of the PD-L1/PD-1 signaling pathway, which promotes anti-viral and anti-tumor immunity, is contingent on interruption of the co-stimulatory signaling cascade received from CD28 (47, 48). However, the interplay between PD-L1/CD80 pathway and CD28 pathway was not investigated. Additionally, the effect of interactions between the PD-L1 signaling pathway and CD28 on alloreactive T cells remains unexplored.

## PD-L1/PD-1 Interaction in Regulating Acute GVHD

The PD-L1-mediated signaling pathway serves as a critical immunological checkpoint of the alloreactive T cell response after HCT in both animal models and humans. Early studies performed by Blazar et al. demonstrated that the PD-L1/PD-1 interactions following HCT are critical for preventing GVHD (20). Following HCT (GVHD murine model: C57BL/6 → B10.BR), the investigators treated HCT recipients with either a blocking PD-L1-IgG2a protein or anti-PD-1 mAb (clone J43). Recipients treated with either method of PD-L1/PD-1 blockade exhibited increased clinical signs of GVHD and mortality compared to control IgG-treated recipients (20). Additionally, recipients of PD-1^−/−^ donor T cells showed increased mortality after HCT compared to recipients of WT donor T cells, indicating a critical role for PD-1 in preventing pathogenic effects of alloreactive T cells (20).

Because PD-1 interacts with a second ligand, PD-L2, to mediate coinhibitory signals to T cells (49), additional work by the Blazar group showed that the PD-L1/PD-1 signaling pathway dominates the PD-L2/PD-1 signaling pathway in the regulation of alloreactive T cell pathogenesis during GVHD (50). Hematopoietic cells upregulate expression of both PD-L2 and PD-L1 after HCT, but only PD-L1 is broadly expressed by parenchymal cells in host GVHD target tissues. Blockade of PD-L1/PD-1 interaction, but not PD-L2/PD-1 interaction can prevent GVHD after HCT (50). Together, these studies identified PD-L1/PD-1 interaction as having a dominant role in regulating alloreactive T cell expansion and pathogenesis to prevent GVHD after HCT.

## PD-L1/CD80 Interaction in Regulating Acute GVHD

Our group has carried out experiments to elucidate how PD-L1/CD80 interaction impacts PD-L1/PD-1 interaction. First, we found that PD-L1/CD80 interaction augments the expansion of donor natural regulatory CD4^+^ T cells (nTregs) after HCT in a minor MHC-mismatched model of GVHD (DBA/2 → BALB/c) (22). Survival of WT donor-derived nTregs after HCT was lower in PD-L1^−/−^ recipients than in WT recipients, which was associated with increased severity of GVHD (22). Moreover, treatment of HCT recipients with either anti-PD-1 mAb or anti-PD-L1 mAb (clone 43H12 which specifically blocks PD-L1/CD80 interaction), demonstrated that PD-L1/CD80 interaction but not PD-L1/PD-1 interaction is responsible for the augmentation of donor nTreg cell survival after HCT (22). Finally, neutralization of IFN-γ, a potent inducer of PD-L1 expression in APCs, led to a reduction in PD-L1 expression by host APCs, a reduction in the number of peripheral nTreg cells and increased severity of GVHD (22).

In a subsequent study, our group also characterized the effect of host parenchymal cell PD-L1 expression on donor alloreactive CD8^+^ T cell pathogenesis in GVHD target tissues after HCT (51). First, we observed that anti-CD3-conditioning prevent induction of acute GVHD, and this effect depends on host-tissue expression of PD-L1. In three murine models of GVHD, we found that increased parenchymal expression of PD-L1 was inversely correlated with the severity of GVHD in the colon, liver, lung and skin (51). Moreover, hepatocyte specific expression of PD-L1-Fc protein induced via hydrodynamic injection of plasmids encoding PD-L1-Fc led to hepatocyte expression of PD-L1, high levels of serum PD-L1, reduced numbers of CD8^+^ T cells infiltrating the liver, and resolution of GVHD (51).

**Figure 1:**
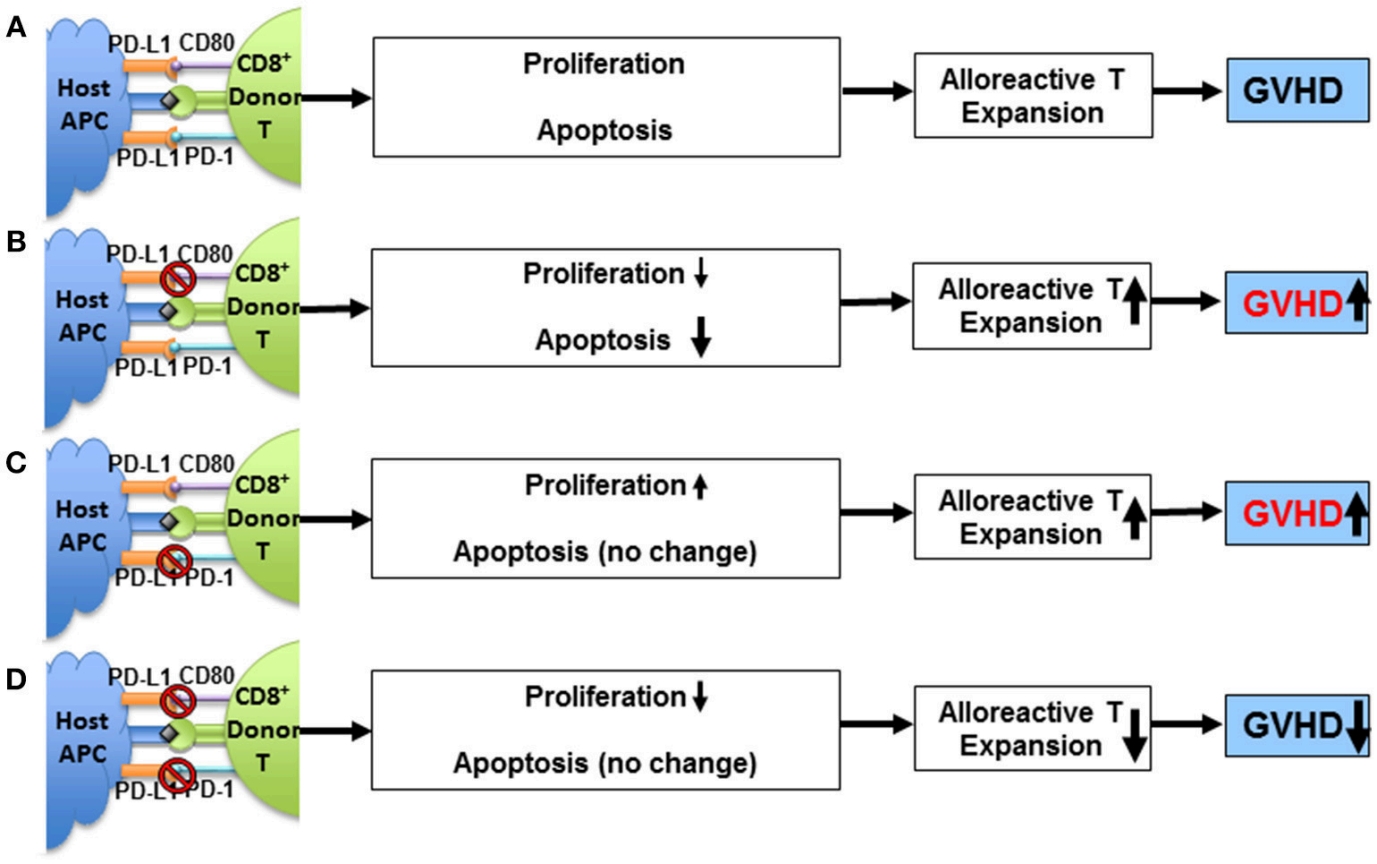
Impact of PD-L1/CD80 interaction on T cell proliferation, PD-1-dependent T cell apoptosis, and GVHD severity. **(A)** Activated alloreactive Tcon cells upregulate expression of PD-1 and CD80 that interact with PD-L1 expressed by host tissue cells (e.g., APCs) after allogeneic HCT. The interactions cannot prevent induction of GVHD. **(B)** Blockade of PD-L1/CD80 interaction reduces alloreactive Tcon cell proliferation and apoptosis, and reduction of apoptosis outweighs reduction of proliferation, resulting in expansion of alloreactive Tcon cells and worsening GVHD. **(C)** In the absence of PD-1 (i.e., lack of PD-L1/PD-1 interaction), PD-L1/CD80 interaction *per se* augments alloreactive Tcon cell proliferation with no impact on apoptosis, resulting in expansion of alloreactive T cells and worsening GVHD. **(D)** In the absence of PD-1, blockade of PD-L1/CD80 interaction reduces Tcon cell proliferation and reduces Tcon expansion and ameliorates GVHD. Adapted from Deng et al. (25).

Although this study demonstrated a clear requirement for PD-L1 expression by parenchymal cells in order to tolerize T cells infiltrating the liver, but the distinct contributions of PD-L1/PD-1 vs. PD-L1/CD80 interactions remained unclear. We subsequently evaluated the separate contributions of the two PD-L1-mediated signaling pathways in regulating GVHD induced by both CD4^+^ and CD8^+^ T cells. As depicted in the diagram (Figure 1), first, using MHC-mismatched C57BL/6 donors and BALB/c recipients, we showed that PD-1^−/−^ donor alloreactive CD4^+^ T cells had decreased proliferation and apoptosis when transferred into PD-L1^−/−^ hosts compared WT hosts (25). PD-L1^−/−^ recipients of PD-1^−/−^ T cells also exhibited a reduction in the severity of GVHD when compared to WT recipients, indicating that the interaction of recipient PD-L1 with CD80 expressed by donor CD4^+^ T cells in the absence of PD-1 increases their expansion, survival and pathogenesis (25). This phenomenon was recapitulated by blocking PD-L1/CD80 interaction on PD-1^−/−^ T cells *in vivo*. In contrast, treatment of allogeneic recipients of PD-1^+/+^ CD4^+^ T cells with anti-PD-L1 43H12 mAb (anti-PD-L1 mAb which specifically blocks PD-L1/CD80 interaction) on day 5 after HCT led to a reduction in proliferation and apoptosis, leading to an augmentation of donor T cell survival and an augmentation of GVHD (25). Finally, *in vivo* expression of PD-L1-Fc protein via hydrodynamic injection augmented PD-1^+/+^ CD4^+^ T cell proliferation and apoptosis leading to an amelioration of GVHD (25). Taken together, these results demonstrate the dual nature of PD-L1/CD80 signaling pathway: PD-L1/CD80 interaction augments T cell proliferation; this interaction also augments activation induced apoptosis mediated by PD-L1/PD-1 interaction. Thus, PD-L1 interaction with PD-1 and CD80 *simultaneously* is required to effectively ameliorate alloreactive T cell-mediated GVHD.

## Differential PD-L1/PD-1 and PD-L1/CD80 Interactions Separate GVL Effects From GVHD

More recent studies investigating the role of PD-L1 expression on donor lymphoid cells and host tissue following HCT have provided further insight into the complexity of PD-L1-mediated signaling pathways. Before HCT, donor T cells express low levels of PD-L1. PD-L1 expression is upregulated on allogeneic but not syngeneic murine donor T cells within 5 days after HCT. PD-L1 is induced within hours after stimulation of human CD4^+^ and CD8^+^ T cells *in vitro* (52). Additionally, genetic ablation of *Pdl1*, the gene that encodes PD-L1, in donor T cells led to a reduction in the severity of GVHD in mice, which was associated with reduced production of proinflammatory cytokines (IFN-γ, TNF-α) and reduced expression of gut homing and chemokine receptors (52). PD-L1^−/−^ donor T cells also exhibited reduced proliferation and yield in lymphoid tissues after HCT, together with increased apoptosis associated with increased expression of FasL and reduced expression of the anti-apoptotic protein Bcl-xL (52).

Our group recently performed a thorough examination of the role of donor T cell expression vs. host parenchymal expression of PD-L1 on the separation of GVHD from GVL activity (26). Using both murine and xeno-GVHD models in which selective depletion of CD4^+^ T cells early after HCT can prevent GVHD while preserving GVL activity, we investigated the differential expression of both PD-L1 and its receptors on donor T cells and in target tissues on the development of GVHD. After CD4^+^ T depletion, IFN-γ produced by donor CD8^+^ T cells upregulated expression of PD-L1 by both host tissues and donor CD8^+^ T cells. The relative expression of PD-1 and CD80 by donor CD8^+^ T cells depends on their location. Donor CD8^+^ T cells in the lymphoid tissues had preferential expression of CD80, while those in GVHD target tissues had preferential expression of PD-1. The dominant interaction of PD-L1 with CD80 in lymphoid tissues promoted donor CD8^+^ T proliferation and survival, thus preserving GVL effects (26). On the other hand, the dominant interaction of PD-L1 with PD-1 on donor CD8^+^ T cells in GVHD-target tissues promoted tolerance through induction of apoptosis, anergy, and exhaustion of CD8^+^ infiltrating T cells (26). Therefore, the outcome of PD-L1-mediated signaling on GVHD and GVL effect depends on the microenvironment and on T cell expression of CD80 and PD-1.

## PD-L1 Pathway Regulation of Alloreactive T Cell Metabolism

As in all cells, immune cells require energy to execute cellular functions, such as survival, proliferation, and cytokine secretion (53). This required energy is provided as adenosine triphosphate (ATP) by several metabolic pathways. The first is glycolysis, which involves the conversion of glucose to pyruvate in the cytosol. The second pathway is the tricarboxylic acid (TCA) cycle (also called the Krebs cycle), which donates electrons to the electron transport chain located in the mitochondria to fuel oxidative phosphorylation (OXPHOS) or respiration. This OXPHOS process generates ATP in the mitochondria. Other substrates, such as fatty acids via β-oxidation (also called fatty acid oxidation [FAO]), can replenish the TCA cycle to fuel OXPHOS. Moreover, the preferential use of glycolysis vs. OXPHOS depends on oxygen availability (53).

T cell proliferation and rapid expansion is a highly dynamic process that taxes intracellular bioenergetics (54). Much like the characteristic “Warburg Effect” observed in malignant cells, the activation and proliferation of T cells requires an immediate switch from a quiescent metabolic state that relies on OXPHOS to an active metabolic state that relies on glycolysis to support rapid flux of ATP, production of biosynthetic intermediates, and synthesis of macromolecules for the process of cell division (55–57). During resolution of the immune response, a subset of T cells augment mitochondrial capacity and revert to multi-substrate OXPHOS, thereby forming memory T cells to provide long-term adaptive immunity (56, 58).

In the context of HCT and GVHD, the kinetic metabolic phenotype of alloreactive T cells remains controversial. Consistent with the canonical paradigm of T cell metabolism during activation and expansion is the recent observation by Rathmell et al. that deletion of Glut1, the primary glucose transporter expressed by T cells, reduces the severity of alloreactive T cell mediated GVHD (59). The reduced severity of GVHD was associated with the failure of Glut1^−/−^ T cells, especially CD4^+^ T cells, to expand *in vivo* (59). Additionally, a thorough follow-up study by the Yu group tracked the metabolic profile of alloreactive T cells after HCT (60). In comparison to syngeneic T cells, alloactivated T cells isolated from recipients with GVHD exhibit a switch from fatty acid oxidation (FAO) in the mitochondria to glycolysis over time after HCT. This change occurred without a subsequent reversion to FAO over time, suggesting that these cells must exhibit an increased reliance on glutaminolysis and the pentose phosphate (PPP) pathway for macromolecule biosynthesis in the absence of the tricarboxylic acid (TCA) cycle (60). In this model, inhibition of glycolysis, either by inhibiting mTorc1 (a key molecular regulator of glycolysis) or 6-phosphofructo-2-kinase/fructose-2,6-biphosphatase 3 (PFKFB3) (a rate limiting step in the glycolytic pathway), reduced the number of alloactivated Th1 cells and ameliorated GVHD (60).

These observations were contradicted by results reported by Byersdorfer et al. who showed that increased Glut1 expression and the switch to glycolysis after alloactivation is merely an *in vitro* phenomenon. Glut1 expression by T cells remained quite static after HCT, while fatty acid uptake and OXPHOS increased in alloactivated T cells by 7 days after HCT (61, 62). Moreover, inhibition of fatty acid uptake by treatment with the irreversible carnitine palmitoyl transerase I (CPT1A; the rate limiting enzyme of fatty acid transport into the mitochondria) inhibitor etomoxir (Etx) induced T cell apoptosis and reduced GVHD severity (61, 63). Finally, an early increase in both glycolysis and OXPHOS in alloactivated T cells has been reported by groups working with Blazar groups (50, 63).

While each of these models has shown some promise in the manipulation of T cell bioenergetics for GVHD prevention, the specific metabolic programs utilized by alloactivated T cells after HCT remain controversial, and a definitive and effective regimen for targeting alloreactive T cell metabolism for the prevention of GVHD remains elusive.

Of interest, Boussiotis et al. recently showed that ligation of PD-1 on human CD4^+^ T cells *in vitro* via PD-L1-Ig stimulation led to upregulation of CPT1A expression with increased fatty acid uptake and enhanced OXPHOS activity, accompanied by downregulation of Glut1 expression and glycolysis via inhibition of the PI3K/AKT/mTOR pathway (39). Consistently, *in vivo* blockade of PD-L1 signaling using anti-PD-L1 mAb (clone 10F.9G2) that blocks both PD-L1/CD80 and PD-L1/PD-1 interactions augmented GVHD and led to an increase in Glut1 expression and glycolysis with a subsequent reduction in mitochondrial respiration (OXPHOS) (64). In contrast, it was also reported that *in vivo* after HCT, alloreactive T cells transplanted into PD-L1^−/−^ recipients exhibited accelerated expansion and pathogenesis; in this situation, absence of host PD-L1 was associated with an increase in both glycolysis and OXPHOS in proliferating donor T cells during expansion (50). Specifically, donor T cells from PD-L1^−/−^ recipients exhibited an increase in Glut1 expression, lactate production (indicative of increased glycolysis), oxygen consumption [indicative of increased utilization of O2 in the electron transport chain (ETC)], mitochondrial membrane potential and ROS production (both indicators of increased mitochondrial respiration) (50). In contrast, the same group reported that PD-L1 deficiency in donor T cells reduces acute GVHD with reduced aerobic glycolysis, oxidative phosphorylation, and fatty acid metabolism in the spleen (52).

The observations with PD-L1^−/−^ recipients suggest that in the absence of host PD-L1, PD-L1 interactions with PD-1 and CD80 among donor cells may modulate donor T cell metabolism. In addition, PD-L1/CD80 interaction may play an important role in augmenting donor cell expansion in lymphoid tissues. We recently demonstrated that PD-L1 expressed by alloactivated donor T cells augment donor T cell expansion in lymphoid tissue through T cell-T cell interaction to augment GVL (26). Importantly, this expansion required donor PD-L1/CD80 interaction (26). Whether and how PD-L1/CD80 interactions among donor T cells affects alloactivated donor T cell metabolism needs to be further addressed.

## Summary

**Figure 2:**
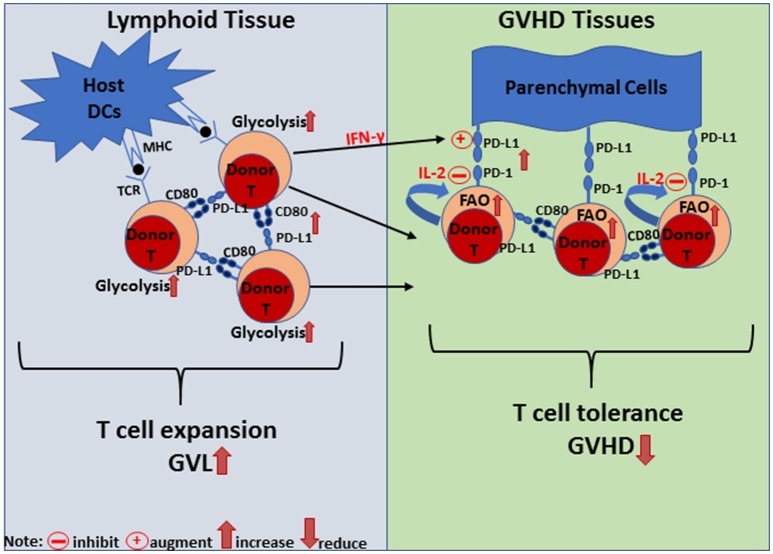
Diagram of PD-L1-mediated signaling regulation of T cell metabolism, proliferation and tolerance in lymphoid and GVHD target tissues. In lymphoid tissue, PD-L1/CD80 interaction is dominant and leads to T cell expansion to promote GVL effects. Signaling either through PD-L1 or CD80 via DC:T or T:T interactions leads to increased glycolysis, proliferation, expansion, and IFN-gamma production. In GVHD target tissues, PD-L1 expressed by parenchymal cells interacts with PD-1 expressed by donor T cells and induces their anergy, exhaustion or apoptosis (depending on the microenvironment) to establish T cell tolerance and prevent GVHD. IFN-gamma produced by donor T cells can augment PD-L1 expression and PD-L1/PD-1 interaction reprograms donor T cells toward a quiescent metabolic phenotype. On the other hand, autocrine or paracrine IL-2 signaling can overcome PD-L1/PD-1 interaction to maintain donor T cell effector function.

Current literature indicates that regulation of GVHD and GVL activity of alloreactive T cells via PD-L1 interaction with PD-1 and CD80 depends on both the tissue compartment and effector cell composition. As depicted in (Figure 2), in the lymphoid tissues, donor cells express higher levels of CD80 and PD-L1 but low levels of PD-1. Here, PD-L1 interaction can augment donor T cell expansion, effector function, and GVL activity. This expansion event may be associated with a PD-L1/CD80 dependent increase in the glycolysis of effector T cells. In contrast, donor T cells express higher levels of PD-1 and lower levels of CD80 in GVHD target tissues, and at the same time, host-tissues express high levels of PD-L1. Here, the simultaneous PD-L1 interaction with PD-1 and CD80 can lead to infiltrating T cell anergy/exhaustion and apoptosis. This PD-L1/PD-1 dominant environment may promote the reversion to a quiescent metabolic phenotype (OXPHOS) in T cells to support reduction in effector T cell function. It should be noted, however, that this mechanism is effective in preventing GVHD only in the absence of donor CD4^+^ T cells or lack of IL-2, because IL-2 from CD4^+^ T cells can help CD8^+^ T cells or help CD4^+^ T cells through autocrine signaling to become resistant against PD-L1-mediated anergy, exhaustion, and apoptosis, perhaps through the promotion of glycolysis (26).

That the 2018 Nobel Prize Award in Medicine was in part awarded to Tasuko Honjo for the discovery of PD-1 highlights the attention that this pathway has achieved abroad and in the clinic for treating cancer. Unfortunately, while much success has been realized with regards to the treatment of cancer with anti-PD-1 mAbs, there are significant side-effects to the non-specific activation of the adaptive immune response (65, 66). Of particular interest to our studies and this review is patient outcome after treatment of relapsed hematological malignancies following HCT (67, 68). Multiple groups have now documented the use of anti-PD-1 mAbs post-HCT for the treatment of relapsed hematological malignancies, such as Hodgkin's lymphoma (67, 69, 70). While outcomes have been favorable regarding cancer-free progression, anti-PD-1 treatment after HCT is associated with the induction of alloimmunity in the form of T cell-mediated acute graft-vs.-host disease (aGVHD) (67, 69). Thus, while the PD-L1/PD-1 pathway is a master regulator of immune-tolerance and can be taken advantage of for the augmentation of anti-tumor immunity, clinical transplantation demonstrates that it is a double-edged sword in that it is also required for the maintenance of T cell tolerance to prevent autoimmunity in cancer patients as well as alloimmunity following HCT. This is consistent with our preclinical observation that PD-L1 checkpoint enforcement, but not blockade, can prevent GVHD after HCT while preserving GVL activity (25, 26).

In future studies, it will be important to determine how PD-L1 interaction with PD-1 or CD80 alone or together regulates donor T cell metabolism. Reagents that regulate T cell metabolism (e.g., 2-DG, ETX, etc.), inhibit IL-2 activity or augment PD-L1/PD1 interactions in GVHD target tissues could tolerize infiltrating T cells while maintaining the function of T cells in lymphoid tissues, such that GVHD could be effectively prevented while strong GVL activity is preserved.

## Author Contributions

KC wrote the manuscript. PM and DZ revised the manuscript.

### Conflict of Interest Statement

The authors declare that the research was conducted in the absence of any commercial or financial relationships that could be construed as a potential conflict of interest.
